# Gut Bacteria and Hydrogen Sulfide: The New Old Players in Circulatory System Homeostasis

**DOI:** 10.3390/molecules21111558

**Published:** 2016-11-17

**Authors:** Lenka Tomasova, Piotr Konopelski, Marcin Ufnal

**Affiliations:** 1 Department of Experimental Physiology and Pathophysiology, Laboratory of Centre for Preclinical Research, Medical University of Warsaw, Warsaw 02 091, Poland; lennytomasova@gmail.com (L.T.); piotr.konopelski@wp.pl (P.K.); 2 Institute of Clinical and Translational Research, Biomedical Research Center, Slovak Academy of Sciences, Bratislava 845 05, Slovakia

**Keywords:** microbiota, gut bacteria, hydrogen sulfide, sulfur, TMAO, indole, cardiovascular diseases, hypertension

## Abstract

Accumulating evidence suggests that gut bacteria play a role in homeostasis of the circulatory system in mammals. First, gut bacteria may affect the nervous control of the circulatory system via the sensory fibres of the enteric nervous system. Second, gut bacteria-derived metabolites may cross the gut-blood barrier and target blood vessels, the heart and other organs involved in the regulation of the circulatory system. A number of studies have shown that hydrogen sulfide (H_2_S) is an important biological mediator in the circulatory system. Thus far, research has focused on the effects of H_2_S enzymatically produced by cardiovascular tissues. However, some recent evidence indicates that H_2_S released in the colon may also contribute to the control of arterial blood pressure. Incidentally, sulfate-reducing bacteria are ubiquitous in mammalian colon, and H_2_S is just one among a number of molecules produced by the gut flora. Other gut bacteria-derived compounds that may affect the circulatory system include methane, nitric oxide, carbon monoxide, trimethylamine or indole. In this paper, we review studies that imply a role of gut microbiota and their metabolites, such as H_2_S, in circulatory system homeostasis.

## 1. Introduction

Increasing evidence suggests that mammalian homeostasis strongly depends on a mutualistic relationship with gut bacteria, and fecal transplantation has recently become a therapeutic option for some intestinal, life-threatening diseases [1]. Interestingly, it has been found that metabolic and cardiovascular diseases, including hypertension, are associated with gut microbiota dysbiosis [2,3,4,5,6,7,8], and some studies suggest that fecal transplantation may also be a therapeutic target in cardiovascular and metabolic diseases [4,9,10].

A number of studies have shown that hydrogen sulfide (H_2_S) and/or the products of its oxidation regulate functions of biological systems, including the circulatory system. Thus far, research has focused on the effects of H_2_S enzymatically produced by mammalian tissues. However, some recent evidence indicates that H_2_S released by bacteria in the colon may also contribute to the control of arterial blood pressure [11,12]. Incidentally, sulfate-reducing bacteria are ubiquitous in mammalian colon, and H_2_S is just one among a number of molecules produced by the gut flora. In this paper, we review studies that imply a role of gut microbiota and gut-bacteria-derived molecules, such as H_2_S, in circulatory system homeostasis.

## 2. Sulfur Bacteria and Life’s Origins

The evolution of Earth is defined by four eons. The Earth was formed in the Hadean eon ~4.6 billion years ago. Life on Earth evolved in the Archean eon (~3.8 billion years ago) in Ferruginous Ocean [13,14]. After the great oxidation event in the Proterozoic eon (~2.3 billion years ago) the concentration of oxygen in the atmosphere increased several times, reaching ~2%. Oceans remained anoxic until the beginning of the Phanerozoic eon ~800 million years ago, when the first modern plants appeared. Theories of life’s origins are trying to answer the question: What was the energy source for driving metabolism in the first organisms? Several lines of evidence suggest that H_2_S was a likely candidate for the first anoxic photosynthetic pathways [15,16,17]. The existence of sulfidogenic organisms in the Archeon is supported by the observation of microfossils-pyrite-associated cells, which are ~3.4 billion years old [18]. First, bacteria were likely sulfur/sulfite disproportionators and sulfite reducers, as these sulfur forms were abundant in the ancient hydrothermal vents. After the great oxidation event in the Proterozoic, sulfate levels in oceans increased, resulting in the domination of sulfate-reduction metabolism [19]. With the raise in the ocean’s oxygen level in the Phanerozoic, the sulfate-reducing bacteria (SRB) were forced to retreat to suboxic and anoxic zones of marine sediments. However, SRB found a suitable anaerobic environment in the gut of mammals.

The modern history of H_2_S is mostly associated with its toxic effects. For the first time, the toxic effects of H_2_S were described in 1713 by Italian physician Bernardino Ramazzini, the father of occupational medicine. Later, H_2_S was used as a chemical weapon in 1916 during World War I. It was only two decades ago, when Abe and Kimura proposed the role of H_2_S as an important biological mediator [20]. Since then, studies have shown that H_2_S is involved in biological signaling in numerous biological systems. Among other biological effects, H_2_S have been reported to exert a hypotensive, cardioprotective and cytoprotective impact [21,22,23,24,25,26].

The research on the regulatory role of H_2_S in the circulatory system thus far has focused mainly on the effects of H_2_S produced by enzymes in the heart, kidneys, vasculature or the brain, while the hemodynamic effect of the gut-bacteria-derived H_2_S has not been evaluated. The biological action of H_2_S produced by the gut microbiota was examined only locally in the gastrointestinal tract [27].

## 3. Gut Bacteria in Mammals: Commensal or Mutualistic Relationship

The mammalian gut is colonized early after birth by bacteria and fungi. The composition of the gut microflora is age, diet and geography dependent [28,29]. Furthermore, the mode of delivery and postnatal feeding shape the microbiota composition, with enriched microflora in vaginal-birth and breastfed babies compared to cesarean-birth and formula-fed babies [30,31]. It is estimated that approximately 10^14^ microbes colonize the healthy mammalian gut. Several bacterial phyla are present in the gut, including Firmicutes, Bacteroidetes, Actinobacteria, Proteobacteria, Verrucomicrobia and Fusobacteria [32].

Gut microbiota plays an important role in the gut motility regulation, dietary fiber and polyphenol digestion, bile acid and steroid transformation, and xenobiotic degradation. Furthermore, it produces a number of various metabolites, such as vitamin K, a key factor in blood clotting. Short-chain fatty acids (acetic, propionic and butyric acid) are formed from undigested carbohydrates complexes as a result of bacterial fermentation carried out by *Lactobacillus* and *Bifidobacterium.* The fatty acids serve as an energy source for colonic intestinal cells and suppress the growth of pathogens by reducing the gut pH [7]. In addition, toxic metabolites (bacteriocins, ammonia, indoles, and phenols) are produced by the gut microbiota inhibiting the colonization of intestines by pathogens. Sulfate and CO_2_ reduction in the gut results in the formation of H_2_S or methane, respectively. Physiological and/or pathological effects of those gut-derived gaseous metabolites remain unclear. However, it was proposed that altered metabolism of gut-derived metabolites may play a role in the pathogenesis of several diseases [33,34,35,36].

### 3.1. Gut Bacteria and the Circulatory System

Gut bacteria may affect the regulation of the circulatory system via at least two pathways. First, gut bacteria and/or their metabolites may stimulate the enteric nervous system. The latter communicates with the brain via afferent sensory fibers. Such a signal may affect the activity of the brain centers involved in the circulatory system control [37]. This pattern of gut-brain axis communication has been previously described for cytokines [38].

Second, gut bacteria and their metabolites may enter the circulation and affect the function of organs and tissues that play a major role in circulatory system homeostasis. The access of gut-derived molecules to the bloodstream is guarded by the gut-blood barrier (GBB), a complex multilayer system which prevents the free passage of compounds between the gut lumen and circulation [39].

### 3.2. The Gut-Blood Barrier

The GBB enables the absorption of nutrients from intestines, and at the same time restricts the passage of pathogens and toxins to the blood [39]. The proper functioning of the GBB may be altered in various diseases [40]. An easier access of gut-derived molecules to the circulation may affect the course of underlying disease and may have a deleterious effect on the entire organism. The integrity and proper functioning of the GBB are preserved by physical and immunological components. The physical barrier is represented by a single layer of epithelial cells which regulate paracellular diffusion and control water and ion absorption. This layer is formed mostly by enterocytes, goblet cells (producing an amorphous polymer-like mucus covering the epithelial cell surface), and immune active Paneth cells. The inner layer of the mucus prevents the adhesion of pathogens to epithelial cells, while the outer layer forms an environment for the commensal bacteria. Interestingly, it has been found that commensal microbiota enhance the integrity of the GBB [2,7]. An immune defense against pathogens, but not against commensal bacteria, is controlled by the system of gut‑associated lymphoid tissue.

Recent studies have pointed to a link between gut microbiota dysbiosis, altered levels of gut-derived metabolites, the GBB dysfunction (GBB leaking), and pathophysiology of various diseases [3,4,5,7,8,10,35,41,42,43,44]. For example, several papers examined the function of the GBB in heart failure (HF), reporting alternations in the GBB permeability and morphology, a reduction in gut blood flow, an increased colonization with specific anaerobes, and higher endotoxin levels in HF patients [45,46,47]. The intestinal blood flow reduction and collagen accumulation were found in patients with advanced HF complicated by cachexia [45,48]. Higher blood endotoxin and cytokine levels were found in edematous HF patients, suggesting that edematous gut wall and epithelial dysfunction resulted in the passage of inflammatory factors into the circulation [46,49].

### 3.3. Gut Bacteria in Cardiovascular and Metabolic Diseases

Recently, the restoration of altered gut microbiota by diet, probiotics, prebiotics or by fecal transplantation has been proposed as a potential therapeutic tool in the treatment of cardiovascular-related problems [4,9,10]. This notion is based on the fact that accumulating evidence shows an association between gut bacteria dysbiosis and cardiovascular and metabolic diseases. For instance, the development of hypertension was recently linked to gut dysbiosis and altered levels of gut-derived metabolites [3]. Yang et al. compared the gut microbiota of normotensive Wistar Kyoto rats and spontaneously hypertensive rats (SHR), and found that SHR rats showed a decreased microbial diversity and lower colonization level of Actinobacteria. Furthermore, the *Firmicute-Bacteroidetes* (F/B) ratio, a marker of gut dysbiosis, have been found to be increased in SHR and in rats with angiotensin II-induced hypertension [50]. In a rat model of obstructive sleep apnea, a high-fat diet resulted in development of hypertension and in a lower butyrate production by gut microbiota. An increase in arterial blood pressure was also observed after transplantation of cecal content from hypertensive obstructive sleep apnea rats into normotensive controls [51]. Mell et al. analyzed differences in bacterial phyla of Dahl salt-sensitive rats that develop hypertension if fed a high-salt diet (S) and Dahl salt‑resistant rats that do not develop hypertension when fed a high-salt diet (R). The S rats showed increased colonization levels of *S24-7* family of *Bacteriodetes* phyla and *Veillonellaceae* family of *Firmicutes* phyla in comparison to the R rats. After intestinal decontamination with antibiotics, the cecal content was transplanted from the S rats to the R rats, and the other way around. Both strains were maintained on a high-salt diet. Surprisingly, the S rats given the R rat microbiota further exacerbated hypertension. This was accompanied by a lower level of fecal bacteria of the *Veillonellaceae* family, increased plasma acetate and heptanoate levels, and a shorter lifespan [5].

It has been well established that there is a strong correlation between high cardiovascular risk, diabetes mellitus and metabolic syndrome. Several lines of evidence suggest that disturbances in gut microbiota composition are also present in metabolic diseases. For example, gut dysbiosis and altered mucosal immunity were found in diabetic patients [8,52]. Children with type 1 diabetes (T1D) showed decreased colonization levels of butyrate-producing bacteria and a negative correlation of the F/B ratio with the glucose level [52]. A metagenome-wide association study of gut microbiota in type 2 diabetes showed gut dysbiosis accompanied by an increase in membrane transport of sugars and branched-chain amino acid, increased methane metabolism, xenobiotic degradation, and sulfate reduction. By contrast, a decrease in the level of bacterial chemotaxis, flagellar assembly, butyrate biosynthesis and metabolism of cofactors and vitamins was found [8]. Studies in animals showed that gut colonization in early life plays an important role in the regulation of fat deposition and development of metabolic syndrome [53]. Furthermore, it was reported that the F/B ratio positively correlates with body weight and is significantly increased in obese people and mice [54,55,56]. On the other hand, some studies found no difference or a decreased F/B ratio in obese patients compared to lean controls [57,58,59]. Further evidence is needed to clarify these discrepancies. The role of gut microbiota in the development of obesity was studied in germ‑free mice. Despite a high-fat, sugar-rich diet, germ-free mice remained lean [60]. Additionally, fecal transplantation from controls to germ-free mice resulted in a 60% increase in body fat and insulin resistance within two weeks [61]. Toll-like receptor 5 (TLR5) expressed by the gut mucosa was suggested to play a role in metabolic syndrome. TLR5-deficient mice showed many features of metabolic syndrome together with gut dysbiosis. Furthermore, transplantation of cecal content from TLR5-deficient mice into wild-type germ-free mice resulted in development of metabolic syndrome [62].

## 4. Gut Bacteria-Derived Molecules and the Circulatory System

Mammalian gut microbiota is a source of a wide range of metabolites. Gut bacteria metabolize carbohydrates, proteins, fat and many other compounds that enter the intestines with food and from hepato-enteric circulation. This includes short-chain fatty acids, alcohols, aldehydes, amines, aromatic derivatives of amino acids (phenols, cresols, indoles), as well as gases, such as H_2_S, methane, NO and CO. Physiological and pathological roles of the gut-derived metabolites are the topic of several reviews [2,33,34,42,63,64]. Here we will focus on the gut-derived molecules that may be involved in the regulation of the circulatory system and in the etiology of cardiovascular diseases.

### 4.1. Hydrogen Sulfide

#### 4.1.1. Gut Bacteria and Hydrogen Sulfide

SRB are ubiquitous members of mammalian colon [65]. The dominant genera are *Desulfovibrio* (*D. piger*, *D. desulfuricans*), *Desulfobacter*, *Desulfobulbus* and *Desulfotomaculu* [19]. Two substrates are essential for SRB to produce H_2_S, i.e., a sulfate and an electron donor for the sulfate reduction. Sulfate-rich diet results in increased growth of *D. piger* and increased H_2_S production in the colon of humans and mice [66,67]. *D. piger* may also utilize sulfated glycans. Since SRB are nonsaccharolytic, they co-colonize *Bacteroides thetaiotaomicron* which liberate sulfate from sulfomucin and mucopolysacharides via sulfatases [66,68]. The presence of *D. piger* positively correlates with the level of the Actinobacterium, *Collinsella aerofaciens*. It is hypothesized that SRB promote the *C. aerofaciens* sugar fermentation by removing the products (H_2_, lactate, formate), which serve as electron donors for the sulfate reduction [66].

SRB represent a nonenzymatic source of H_2_S in the mammals gut. The second source is enzymatic generation performed by either gut bacteria or colonic tissues. Several anaerobic bacterial strains (*Escherichia coli*, *Salmonella enterica*, *Clostridia* and *Enterobacter aerogenes)* convert cysteine to H_2_S, pyruvate and ammonia by cysteine desulfhydrase [69,70]. In addition, gut bacteria may produce H_2_S by sulfite reduction. Sulfite reductase is present in many species such as *E. coli*, *Salmonella*, *Enterobacter*, *Klebsiella*, *Bacillus*, *Staphylococcus*, *Corynebacterium*, and *Rhodococcus* [71]. The generation or utilization of H_2_S in reactions catalyzed by sulfite reductase is dependent on redox potential [72]. Finally, mammalian tissues can synthesize H_2_S from l‑cysteine and l‑homocysteine in reactions catalyzed by cystathionine beta‑synthase (CBS), cystathionine gamma‑lyase (CSE) and 3‑mercaptopyruvate sulfurtransferase (3-MST). CSE and CBS were reported to be present in the gastrointestinal tract of rodents and humans [73,74,75,76], while the CSE seems to be a major source of the gut H_2_S generation [77].

The total sulfide concentration in the luminal content of the large intestine has been reported to be in the range of 0.2–3.4 mmol/L in humans [78], rats [79] and mice [80]. It needs to be stressed that the feces of humans and rodents have a large binding capacity, and less than 8% of total sulfide was found to be in a free form [79,81]. Interestingly, colonic epithelial cells are more efficient in converting sulfide into thiosulfate than other tissues [82]. In the study of Levitt et al., the analysis of cecal venous blood after intracecal infusion of radioactive H_2_S in rats revealed that all absorbed H_2_S had been oxidized to thiosulfate [83].

The proportion of H_2_S synthesis derived from bacteria and colonic tissue was examined by Flannigan et al. [84]. They have found that fecal samples of germ-free mice contained half of H_2_S in comparison to feces of controls. Furthermore, it was shown that the absence of vitamin B_6_, a CSE and CBS cofactor, in the diet resulted in a 50% reduction of fecal H_2_S. The deficiency of vitamin B_6_ in the diet significantly reduced fecal H_2_S levels, likely due to the inhibition of enzymatic H_2_S synthesis in colonic tissues. Interestingly, after six weeks of a vitamin B_6_-deficient diet, the fecal H_2_S levels returned to the same levels as in controls. This suggests that the H_2_S generation in the gut of germ-free mice was shifted towards nonenzymatic pathways by increasing the SRB activity [84].

Shen et al. showed that germ-free mice exhibited decreased levels of free H_2_S in inferior vena cava blood plasma and in gastrointestinal tissues, and reduced bound sulfane sulfur levels in plasma, adipose tissue and lung tissue. Furthermore, the activity of CSE was significantly lower, whereas the level of l‑cysteine, a substrate for H_2_S synthesis, was markedly elevated in gastrointestinal and extraintestinal tissues (aorta, liver, and kidney) of germ-free mice compared to control mice [12].

In our studies, rats treated with neomycin (an antibiotic that does not cross the GBB and is used for intestinal decontamination in liver failure patients to reduce microbiota-produced NH_3_) exhibited significantly decreased levels of thiosulfate and sulfane sulfur, products of H_2_S oxidation, in portal vein blood plasma but not in peripheral blood plasma [11]. Furthermore, we found that intracolonic administration of Na_2_S (a H_2_S donor) increases portal blood levels of thiosulfate and sulfane sulfur, while no such significant effect was observed in peripheral blood. These findings imply that the liver may buffer the thiosulfate and sulfane sulfur pools in the organism, and suggest that systemic effects of colon-derived H_2_S and/or its derivatives may be in part due to some liver-dependent mechanisms [11].

Several studies investigated the effect of intestinal H_2_S on gut functions. On the one hand, it has been suggested that high colonic H_2_S levels may be responsible for colonic inflammation and cancer [73,85]. On the other hand, recent studies suggest that colonic epithelial cells are well-adapted to the H_2_S-rich environment, and that H_2_S plays a beneficial role in the protection of the GBB [27,86,87]. First, it has been proposed that H_2_S may serve as an energy source for colonic epithelial cells, since the oxidation of gut H_2_S results in ATP formation [87]. Second, Motta et al. reported that H_2_S promotes colonic mucus production and integrity of microbiota biofilms [86]. Third, gut dysbiosis induced by chronic administration of nonsteroidal anti‑inflammatory drugs was reversed by exogenous H_2_S [88].

#### 4.1.2. Hydrogen Sulfide in the Circulatory System

**Figure 1:**
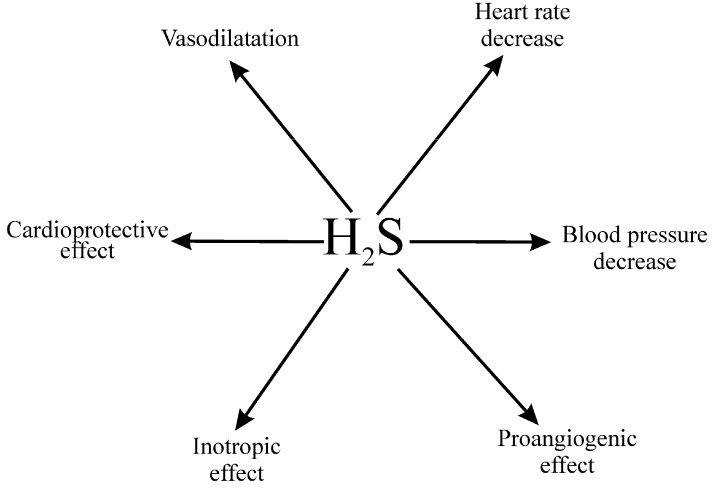
Major cardiovascular effects of hydrogen sulfide donors (H_2_S, ref.: [11,25,26,89,90,91,92,93,95,97]).

Many studies describe the effects of H_2_S in the circulatory system, which have been thoroughly reviewed elsewhere [89,90,91,92,93]. In short, H_2_S is synthetized in various tissues involved in circulatory system homeostasis, including the heart, blood vessels, kidneys and the brain, by CSE and CBS. Depending on the methods employed, the estimated concentration of H_2_S in the blood and other tissues has been reported to be within the range of 30 and 200 µmol/L [93]. However, recent studies suggest that physiological concentration of H_2_S in cardiovascular tissues is in nanomolar range [94], in contrast to millimolar concentrations in the intestines [78,79,80]. Administration of H_2_S donors produces a decrease in arterial blood pressure, which appears to depend mostly on vasodilation, but the effect may be dose- and species-specific [11,26,89,90,91,92,93,94,95]. The mechanisms behind the H_2_S-mediated vasodilation are not clear. One of the postulated theories is an opening of ATP-sensitive potassium channels [89]. In addition to its hemodynamic effects, H_2_S has been shown to produce cardioprotective, proangiogenic and cytoprotective effects, and disturbances in H_2_S homeostasis have been suggested to be involved in the etiology of cardiovascular and metabolic diseases [90,91,95,96,97], (Figure 1). Therefore, it is not surprising that H_2_S donors have attracted a great deal of attention as potential drugs. Although H_2_S-based balneotherapy has been practiced for centuries, there is still no solid evidence to support the use of H_2_S donors in clinical practice. At present, experimental and clinical studies are being performed to evaluate the therapeutic potential of several H_2_S donors, in particular in cardiovascular and gastrointestinal diseases [97].

It needs to be noted that biological effects of H_2_S may depend on its interaction with NO, and formation of new molecules, such as *S*-nitrosothiols. Interactions of NO and H_2_S have been elegantly reviewed elsewhere [89]. Furthermore, H_2_S is rapidly oxidized into thiosulfates and other products [11,79,81,82,83]. It is likely that both H_2_S and products of its oxidation contribute to the regulation of the circulatory system.

#### 4.1.3. Cardiovascular Effects of the Gut-Derived Hydrogen Sulfide

**Figure 2:**
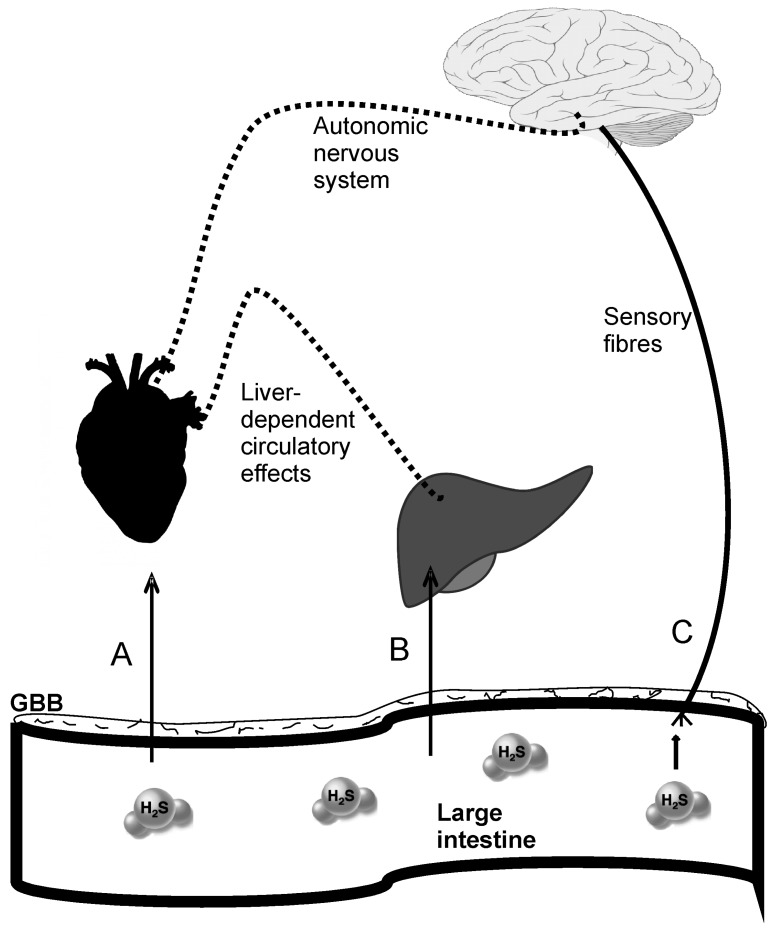
Postulated pathways of cardiovascular actions of gut-bacteria-derived hydrogen sulfide and its derivatives (H_2_S). (**A**) H_2_S crosses the gut-blood barrier (GBB), bypasses the liver (*rectal plexuses*)*,* and targets the heart and blood vessels; (**B**) H_2_S crosses the GBB and affects liver functions associated with the circulatory system homeostasis; (**C**) H_2_S stimulates sensory fibers of the enteric nervous system that project to the brain centers controlling the circulatory system via the autonomic nervous system.

The studies on the role of H_2_S in the circulatory system have thus far focused on the effects of H_2_S produced enzymatically by various tissues. Strikingly, although colon microbiota represents the greatest source of H_2_S in the body, the effects of colon-derived H_2_S on the circulatory system have not been studied. In our laboratory, we examined the effects of increased availability of H_2_S in the colon on rat hemodynamics. Intracolonic administration of Na_2_S (a H_2_S donor) exerted a potent, long‑lasting hypotensive effect which persisted several times longer than previously reported after parenteral infusions (>90 min). Interestingly, hypertensive rats showed a more pronounced decrease in arterial blood pressure than normotensive rats. Besides, rats treated with neomycin showed significantly decreased levels of thiosulfate and sulfane sulfur, and a tendency for greater hypotensive response to Na_2_S. These data suggest that the gut-derived H_2_S may produce systemic effects, and that changes in colonic H_2_S homeostasis may be associated with hypertension. In our study, the hypotensive effect was most probably due to peripheral vasodilation and a decrease in heart rate. In contrast, local changes in intestinal blood flow were not a likely cause of the H_2_S-dependent hypotension. The hemodynamic effects of intracolonic H_2_S donor were accompanied by increases in portal but not peripheral blood levels of H_2_S oxidation products [11]. Therefore, it seems that the systemic effects of the gut H_2_S were produced by either some liver-dependent mechanisms or by the effects of colonic H_2_S on the enteric nervous system (Figure 2). All in all, our findings support previous evidence on the hypotensive effect of H_2_S and/or its derivatives, and at the same time provide new data implying a role of gut-bacteria-derived H_2_S in blood pressure control.

### 4.2. Nitric Oxide

Nitric oxide (NO) is one of the most studied biological transmitters. It plays a significant role in numerous biological systems, including the circulatory system. Intriguingly, as mentioned above, some evidence suggests that NO interacts with H_2_S, and that this interaction may determine the final biological effects of both gaseous transmitters [89,98,99].

Several pathways of NO formation in the mammalian gastrointestinal tract have been proposed. The first is the nitrate-nitrite-NO pathway. Nitrate is reduced by commensal mouth bacteria to nitrite [100], which is further reduced by gut bacteria, either by nonenzymatic acidic reduction [101] or by nitrite reductases [102]. Finally, gut mucosa express NO synthase which synthetize NO’s converting of l-arginine to l-citrullin [103]. It has also been found that the probiotic strains *Lactobacillus* and *Bifidobacterium* play a role in the intestinal production of NO by decreasing gut pH which increases nonenzymatic nitrite reduction [104]. In contrast, *Desulfovibrio vulgaris* convert NO to nitrates [105].

The role of NO in physiology and pathology of the gastrointestinal system [106,107,108,109] and the cardiovascular system [110,111,112,113,114] was reviewed elsewhere. However, the role of the gut-derived NO in the circulatory system homeostasis remains obscure. Similar to H_2_S, the circulatory effects of NO were mostly evaluated in the context of its enzymatic production by various tissues. However, Briskey et al. proposed a role of nitrification/denitrification pathway in the regulation of mammalian homeostasis [115]. Dependent on the redox state, *E. coli* and *Lactobacillus plantarum* reduce nitrites to ammonia (denitrification) [116] or ammonia is oxidized by *Nitrosomonas* back to nitrite (nitrification) [117]. Dysregulation of this pathway can lead to pathological accumulation of gut-derived ammonia or nitrite, gut dysbiosis and related cardiovascular problems [118,119,120]. A limitation of this hypothesis is that the presence of ammonia-oxidizing bacteria and archaea is described only in soil, water and plants [121].

### 4.3. Carbon Monoxide

A number of studies have shown the importance of the roles played by carbon monoxide (CO) in the circulatory system. Similar to NO and H_2_S, CO has been found to exert vasorelaxant and cardiac protection effects [122,123]. CO is produced in a reaction catalyzed by the enzyme heme oxygenase (HO). Inducible HO (HO-1) and constitutive HO (HO-2) are mostly recognized for endogenous CO production in mammalian tissues. In the gastrointestinal system CO may be produced by gut mucosa which expresses HO-1. Furthermore, Onyiah et al. reported that also gut microbiota (*E. coli*) express HO homologs [124] and induce colonic expression of HO-1 in mice [125]. The possible effects of gut-derived CO on systemic circulation remain to be elucidated.

### 4.4. Methane

The mammalian gut is colonized by methanogenic archeaea: Methanobacteriales, Methanococcales, Methanomicrobiales, Methanosarcinales, Methanopyrales, Methanocellales, Methanomassiliicoccales [2]. In human gut, the dominant methanogen is Methaninobrevibacter smithii. According to substrate utilization, there are three types of methanogens [126]: (i) The most common are hydrogenotrophs, which use H_2_ or formate as an electron donor for CO_2_ reduction [127]; (ii) Methylotrophs convert methylated compounds (methanol, methylamines and methyl-sulfides) by substrate-specific methyltransferases into methane [128]; and (iii) Acetotrophs produce methane utilizing acetate [129]. Methane may also be produced by certain Clostiridium and Bacteroides species [130].

Interestingly, sulfate reduction and methanogenesis compete for the mutual substrate, which is H_2_. The methanogenesis/sulfate reduction ratio is dependent on substrate availability, thermodynamics and pH. In human colon, the ratio is in the favor of methanogenesis, due to neutral pH of stool and low sulfate levels in diet. However, in certain conditions, such as high availability of sulfate substrates in a diet (bread, beer, wine) and hypochlorhydria, sulfate reduction may become the major process [131,132].

The physiological levels of methane in the mammalian organism have not yet been determined [133]. Breath tests show that 30%–60% of healthy individuals produce gaseous methane [134,135,136]. As in the case of the gut-derived H_2_S, methane metabolism was mostly studied in association with gastrointestinal problems, such as constipation, diarrhea and irritable bowel syndrome [64,137,138,139,140,141,142].

Some studies suggest that altered methane metabolism may also play a role in cardiovascular and metabolic diseases. Methanogen growth was positively correlated with the development of obesity and diabetes [8,143,144]. Furthermore, it has been found that exogenous methane may reduce oxidative and nitrosative stress in animal model of ischemia‑reperfusion injury [63].

### 4.5. Trimethylamine

The production and utilization of methylamines, in particular trimethylamines, by gut bacteria has recently attracted a lot of attention. This is because several clinical studies showed a positive correlation between elevated plasma levels of trimethylamine *N*-oxide (TMAO) and an increased risk for adverse cardiovascular events. Methylamines are bacterial products of dietary choline and carnitine [42]. Several bacterial species were reported to participate in intestinal metabolism of methylamines including: *Anaerococcus hydrogenalis*, *Clostridium asparagiforme*, *Clostridium hathewayi*, *Clostridium sporogenes*, *Escherichia fergusonii*, *Proteus penneri*, *Providencia rettgeri* and *Edwardsiella tarda* [145]. The blood concentration of TMAO was reported to be in the range of 0.5–5 μmol/L in healthy individuals and rodents [42].

Some studies suggest that TMAO may be a causative link between the diet, gut bacteria and cardiovascular diseases. For example, it has been found that TMAO augments heart failure in mice, plays a role in the development of atherosclerosis by modulating cholesterol and sterol metabolism [146], and prolongs the hypertensive effect of angiotensin II [147], a key hormone in circulatory system homeostasis. On the other hand, it has been reported that vascular injury and oxidative stress were reduced after l-carnitine rich diet, which resulted in elevated TMA and TMAO plasma levels [148]. In addition, an increase in TMAO blood level in rats from 0.6 to 60 μmol/L for two weeks did not produce any apparent toxic effect [147]. Finally, it is worth noting that high concentrations of TMAO (100 μmol/L and higher) are found in saltwater fish, the consumption of which has been considered to have a beneficial effect on the circulatory system [42]. Further studies are needed to assess the physiological and pathological importance of TMAO in humans.

### 4.6. Indole

Various bacteria, more than 85 species, can metabolize trypthophane and form indole. For example, the conversion of tryptophan into indole, pyruvate and ammonia is catalyzed by tryptophanase in *E. coli* [149]. Indole was detected in mammalian feces [150,151,152], and gut bacteria produce indole presumably by enzymes homologous to tryptophanases [33]. In the gut, indole is either oxidized by bacterial oxygenases or by cytochrome P450 to form indoxyl, which is further sulfonated in the liver to indoxyl sulfate (IS) and excreted with urine [149].

Indole production was studied mostly in association with the regulation of bacterial physiology. It was reported that indole regulates spore formation, drug resistance, virulence, plasmid stability, and biofilm formation in several bacteria [149,153]. The role of indole in the regulation of mammalian homeostasis remains unclear. Cardiorenal syndrome, a combination of cardiovascular and kidney disorders, chronic kidney failure, and vascular remodeling have been found to be positively associated with increased concentration of circulating IS [154,155,156,157]. Furthermore, IS blood level may serve as a predictor of cardiovascular events and mortality in chronic kidney patients [155,156]. Some experimental studies in rats suggest that a decrease in IS level inhibits the progression of cardiomyopathy and chronic kidney failure [158,159,160]. Other studies imply that indole may protect the GBB integrity [161] and have anti‑inflammatory properties [161,162].

### 4.7. Ammonia

A great pool of ammonia (NH_3_) is formed in the mammalian gut by several bacterial species and gastrointestinal tissues [36]. In fact, the degradation of urea by gut microbiota ureases (~7 g/day) is the source of around 50% of total NH_3_ in the body [163]. The NH_3_ production rate in the human gut is 4–10 g/day [36]. Unbound NH_3_ is either excreted with feces (~5–25 μg/g) [34] or retransformed into amino acids by gut microbiota, or absorbed through the GBB. The plasmatic concentration of free NH_3_ in healthy individuals is ~35 μmol/L [36]. Circulating NH_3_ can be either converted into urea or glutamine in the liver or excreted with urine (2–3 mg/day) [163].

Liver disorders associated with hyperammonemia and related neurotoxic effects are well described, and patients with liver failure are often treated with antibiotics, such as neomycin, to decontaminate the intestines and decrease bacterial production of NH_3_. Interestingly, there is some evidence that NH_3_ may affect the control of the circulatory system. For example, a positive inotropic effect of NH_3_ on isolated rat hearts was reported [164]. Moreover, it has been found that NH_3_ inhalation in healthy adults results in cerebrovascular vasodilatation without affecting the arterial blood pressure [165]. Finally, patients with HF show increased plasma levels of NH_3_ [119,120].

## 5. Conclusions

Several lines of evidence suggest that gut bacteria may affect the functioning of the circulatory system. Trimethylamine *N*-oxide (TMAO), a gut-bacteria-derived molecule, has recently emerged as a new diagnostic marker of increased cardiovascular risk, and gut dysbiosis has been found in cardiovascular and metabolic diseases. Sulfate-reducing bacteria are abundant in the mammalian colon, producing significant amounts of sulfur compounds, including H_2_S. Despite a large number of studies on H_2_S in the circulatory system, there is scant data on the effects of gut-derived sulfur compounds. Further research on gut sulfate-reducing bacteria and their products is needed as they may become a therapeutic target in cardiovascular diseases.
